# Dysferlin Deficiency Results in Myofiber-Type Specific Differences in Abundances of Calcium-Handling and Glycogen Metabolism Proteins

**DOI:** 10.3390/ijms24010076

**Published:** 2022-12-21

**Authors:** Erin M. Lloyd, Gavin J. Pinniger, Miranda D. Grounds, Robyn M. Murphy

**Affiliations:** 1 Department of Anatomy, Physiology and Human Biology, School of Human Sciences, The University of Western Australia, Perth, WA 6009, Australia; 2 Department of Biochemistry and Chemistry, School of Agriculture, Biomedicine and Environment, La Trobe University, Melbourne, VIC 3086, Australia

**Keywords:** dysferlinopathy, BLAJ mouse, myofiber-types, skeletal muscle contraction, calcium-handling proteins, glucose/glycogen metabolism proteins

## Abstract

Dysferlinopathies are a clinically heterogeneous group of muscular dystrophies caused by a genetic deficiency of the membrane-associated protein dysferlin, which usually manifest post-growth in young adults. The disease is characterized by progressive skeletal muscle wasting in the limb-girdle and limbs, inflammation, accumulation of lipid droplets in slow-twitch myofibers and, in later stages, replacement of muscles by adipose tissue. Previously we reported myofiber-type specific differences in muscle contractile function of 10-month-old dysferlin-deficient BLAJ mice that could not be fully accounted for by altered myofiber-type composition. In order to further investigate these findings, we examined the impact of dysferlin deficiency on the abundance of calcium (Ca^2+^) handling and glucose/glycogen metabolism-related proteins in predominantly slow-twitch, oxidative soleus and fast-twitch, glycolytic extensor digitorum longus (EDL) muscles of 10-month-old wild-type (WT) C57BL/6J and dysferlin-deficient BLAJ male mice. Additionally, we compared the Ca^2+^ activation properties of isolated slow- and fast-twitch myofibers from 3-month-old WT and BLAJ male mice. Differences were observed for some Ca^2+^ handling and glucose/glycogen metabolism-related protein levels between BLAJ soleus and EDL muscles (compared with WT) that may contribute to the previously reported differences in function in these BLAJ muscles. Dysferlin deficiency did not impact glycogen content of whole muscles nor Ca^2+^ activation of the myofilaments, although soleus muscle from 10-month-old BLAJ mice had more glycogen than EDL muscles. These results demonstrate a further impact of dysferlin deficiency on proteins associated with excitation-contraction coupling and glycogen metabolism in skeletal muscles, potentially contributing to altered contractile function in dysferlinopathy.

## 1. Introduction

Dysferlinopathies, are a clinically heterogeneous group of neuromuscular disorders that arise from mutations in the dysferlin gene, resulting in reduced expression of functional dysferlin protein (reviewed by Amato et al. [1]; Cárdenas et al. [2]). Dysferlinopathies are characterized by progressive skeletal muscle weakness and muscle wasting [3,4], with symptoms manifesting usually post-growth (in young adults). The pathologies typically manifest initially as limb-girdle muscular dystrophy type R2 dysferlin-related (LGMDR2; previously known as LGMD2B [5]) or Miyoshi myopathy, with initial weakness typically in the proximal limb girdle or distal muscles, respectively [6], although gradations across phenotypes are now more widely recognized [7]. Studies in dysferlin-deficient (dysf^−/−^) mouse models (including the BLAJ (B6.A-Dysf^prmd^/GeneJ), A/J (A/J^dysf−/−^), and SJL/J (SJL/J^dysf−/−^) mice; discussed in van Putten et al. [8]) and humans show early accumulation of lipid droplets (steatosis) in slow-twitch myofibers [9,10,11], altered lipid metabolism and lipidomics in young adult BLAJ mice [12], inflammation [13] and, in later stages, replacement of muscles by adipocytes [1,9,10,11,14,15].

Dysferlin is a calcium (Ca^2+^)-binding protein highly expressed in skeletal muscle and localized to the transverse (T)-tubules (especially during development) and sarcolemma (predominant in mature myofibers), but also present in many other cell types including macrophages, neutrophils, adipocytes, and vascular endothelial cells [9,16]. Notably, dysferlin plays an important role in vesicle trafficking and fusion [17,18] and skeletal muscle T-tubule formation [19,20], where dysf^−/−^ skeletal muscle is characterized by abnormally configured T-tubules [21]. In addition, dysferlin is implicated in Ca^2+^ handling associated with excitation-contraction (EC) coupling [16,21], including interacting with Ca^2+^-handling proteins in the T-tubules [19,22]. Hence, it is hypothesized that disruption of Ca^2+^ handling and EC coupling in dysf^−/−^ muscle may contribute to muscle weakness and fatigability that is observed in this disease [20,23]. However, these observations need to be reconciled with the fact that patients with dysferlin deficiency appear to have no problem with muscle formation and function for many years until late adolescence when the disease phenotype is typically observed.

Furthermore, the accumulation of lipid within muscles suggests a metabolic contribution to the disease pathology. Lipidomic studies in young BLAJ mice showed marked changes in lipid metabolism and lipid composition of dysf^−/−^ muscles [12]. Specifically, we reported that three-month-old BLAJ soleus muscles had increased triglyceride storage compared with normal wild-type (WT) C57BL/6J controls, while triglyceride levels were unchanged in the ‘fast/mixed’ BLAJ quadriceps, one of the earliest muscles to show severe dystropathology in mice [12]. Moreover, using metabolomic analysis, Schoewel et al. [24] reported impaired glycolytic metabolism in dysf^−/−^ mice and primary human myoblasts from dysferlinopathy patients, specifically decreased muscle glycogen and impaired glucose utilization, which may be compensated for by the presence of intermyofibrillar lipid. However, this work was not published further than the abstract noted, and absolute values were not reported, thus further investigation is warranted.

Due to the marked differences in contractile and metabolic properties of slow- and fast-twitch myofibers, and since many muscles are a complex mixture of different myofiber types [25], we compared in mice the functional properties [26] of the predominantly slow soleus muscle composed of about 37% slow type 1 and 63% intermediate (2A/2X) myofibers, with the predominantly fast extensor digitorum longus (EDL) muscle composed of about 34% intermediate and 66% fast type 2B myofibers [27]. Our study showed myofiber-type specific differences in contractile function of soleus and EDL muscles from dysf^−/−^ BLAJ mice (compared with WT) aged 10 months, including faster contraction times and delayed fatigue recovery for the soleus, and slower contraction times for the EDL muscles [26]: these functional differences could not be fully accounted for by altered myofiber-type composition, implicating possible alterations in EC coupling in dysf^−/−^ muscle. To further investigate the factors contributing to these functional changes, we examined the impact of chronic dysferlin deficiency on the abundance of selected Ca^2+^ handling and glucose/glycogen metabolism-related proteins, and glycogen in soleus and EDL muscles from WT and BLAJ mice aged 10 months, and also the intrinsic Ca^2+^-activation properties of the contractile apparatus (i.e., myofilaments) in individual slow- and fast-twitch myofibers isolated from muscles of WT and BLAJ mice aged three months, which is prior to the manifestation of the disease.

## 2. Results

### 2.1. Phenotype

For three-month-old mice, there was no significant difference between WT and BLAJ mice for body mass or myofiber cross-sectional area (Table 1). For 10-month-old mice, as reported previously [26], there were no differences in body mass between the male WT and BLAJ mice. However, the BLAJ soleus (normalized to body mass) was heavier compared with the WT (*p* < 0.001), while EDL mass was unaffected.

### 2.2. Quantification of Proteins Related to Ca^2+^ Handling and Glucose/Glycogen Metabolism in Soleus and EDL Muscles of Mice Aged 10 Months

**Figure 1:**
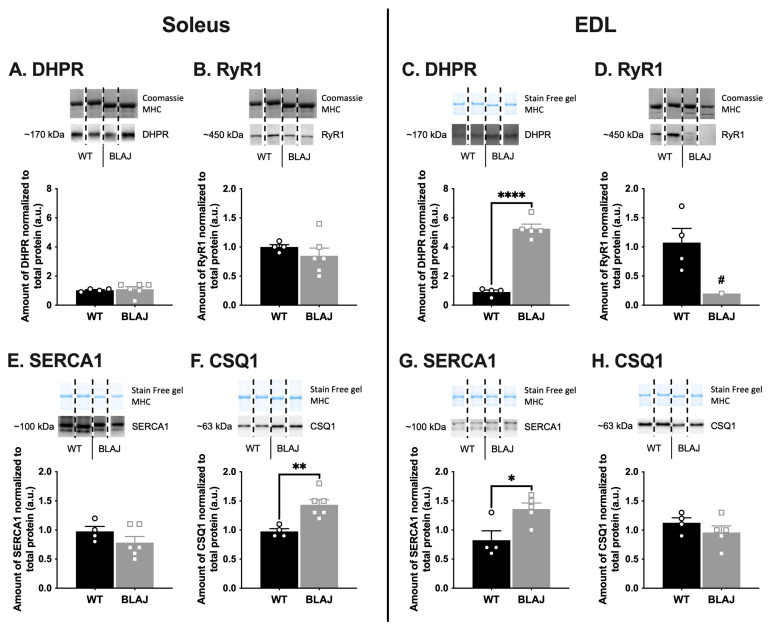
The levels of proteins associated with calcium (Ca^2+^) handling in soleus and EDL muscles, of WT compared with dysferlin-deficient BLAJ male mice aged 10 months. Whole muscle homogenates analyzed by Western blot. (**A**,**C**) Dihydropyridine receptor (DHPR); (**B**,**D**) Ryanodine receptor 1 (RyR1); (**E**,**G**) Sarco/endoplasmic reticulum Ca^2+^-ATPase 1 (SERCA1); (**F**,**H**) Calsequestrin 1 (CSQ1). Data analyzed by Student’s *t*-test: *, **, **** BLAJ vs. WT (*p* < 0.05, 0.01, 0.0001, respectively). **#** RyR1 levels were reduced and undetectable in five of six BLAJ EDL muscles analyzed resulting in *n* = 1 for this group, hence statistical comparisons were not appropriate to be reported for these data. Data are presented as individual values (WT as circles, BLAJ as squares) and mean ± SEM (*n* = 1–6), with representative Western blots where non-contiguous lanes from the same gel are separated by black dashed lines.

**Figure 2:**
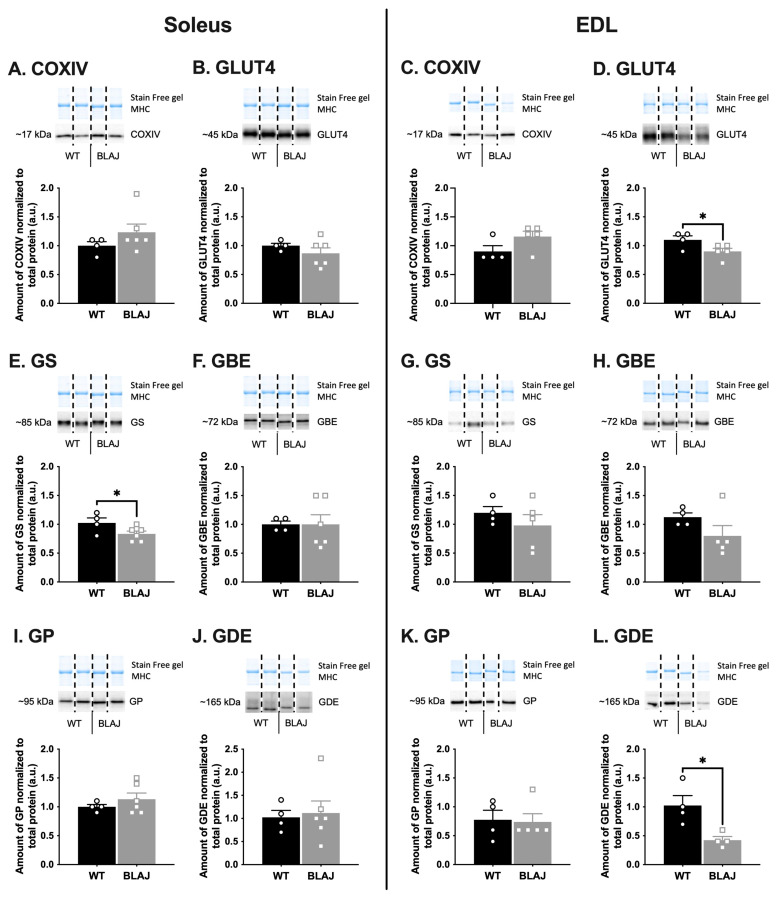
The levels of proteins associated with glucose/glycogen metabolism in soleus and EDL muscles, of WT compared with dysferlin-deficient BLAJ male mice aged 10 months. Whole muscle homogenates analyzed by Western blot. (**A**,**C**) Cytochrome c oxidase subunit IV (COXIV). (**B**,**D**) Glucose transporter type 4 (GLUT4); (**E**,**G**) Glycogen synthase (GS); (**F**,**H**) Glycogen branching enzyme (GBE); (**I**,**K**) Glycogen phosphorylase (GP); (**J**,**L**) Glycogen debranching enzyme (GDE). Data analyzed by Student’s *t*-test: * BLAJ vs WT (*p* < 0.05). Data are presented as individual values (WT as circles, BLAJ as squares) and mean ± SEM (*n* = 4–6), with representative Western blots where non-contiguous lanes from the same gel are separated by black dashed lines.

The abundance of key proteins associated with EC coupling (Figure 1) and glucose/glycogen metabolism (Figure 2) were quantified to determine if these might be altered in BLAJ compared with WT muscles, using (slow) soleus and (fast) EDL muscles of male mice aged 10 months. This study used the contralateral muscles from the same individual mice where we demonstrated functional differences between these muscles [26]. Marked myofiber-type specific differences between WT and BLAJ muscles were evident for several proteins. Compared with WT, the main differences in BLAJ (slow) soleus were increased abundance of calsequestrin 1 (CSQ1; Figure 1F) and decreased glycogen synthase (GS; Figure 2E) compared with WT (*p* < 0.05). Whereas, for BLAJ (fast) EDL muscles there were increased levels of dihydropyridine receptor (DHPR; Figure 1C) and sarco/endoplasmic reticulum Ca^2+^-ATPase 1 (SERCA1; Figure 1G), and decreased glucose transporter type 4 (GLUT4; Figure 2D) and glycogen debranching enzyme (GDE; Figure 2L; *p* < 0.05). Notably, for BLAJ EDL muscles the abundance of ryanodine receptor 1 (RyR1) was also reduced (see EDL in Figure 1D), where RyR1 levels were below the level of detection for five of the six muscles analyzed.

### 2.3. Glycogen Content

**Figure 3:**
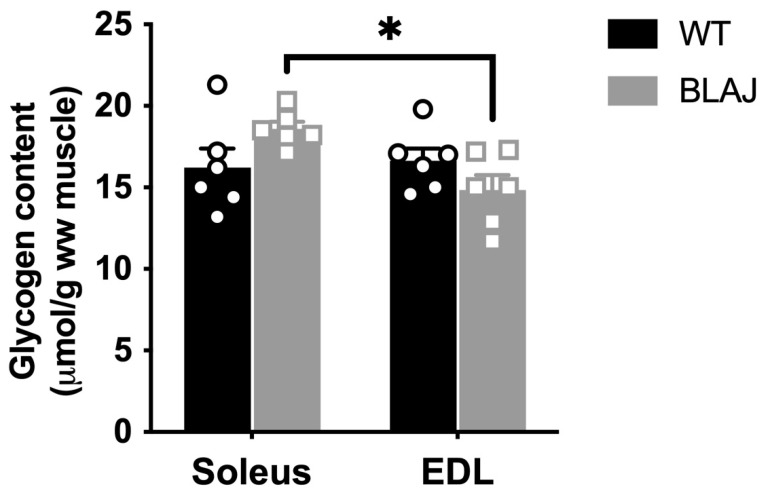
Glycogen content in soleus and EDL muscles of WT compared with dysferlin-deficient BLAJ male mice aged 10 months. Whole muscle homogenates analyzed for glycogen content. Data analyzed by two-way Analysis of Variance (ANOVA) with Holm-Bonferroni multiple comparisons: * BLAJ soleus vs BLAJ EDL (*p* < 0.05). Data are presented as individual values (WT as circles, BLAJ as squares) and mean ± SEM (*n* = 6).

Glycogen content did not differ between WT and BLAJ for either soleus or EDL muscles (Figure 3). When assessing the impact of both dysferlin deficiency and muscle-type via two-way Analysis of Variance (ANOVA), there was an interaction effect of mouse strain and muscle-type (*p* < 0.05), where glycogen content was higher in soleus compared to EDL muscles from BLAJ mice (*p* < 0.05), but similar between EDL and soleus muscles from WT mice.

### 2.4. Ca^2+^ Activation of the Contractile Apparatus from Individual Slow- and Fast-Twitch Myofibers from Mice Aged Three Months

In order to examine the intrinsic Ca^2+^-activation properties of slow- and fast-twitch contractile apparatus, individual myofibers were isolated from soleus and EDL muscles of WT and BLAJ mice aged three months, chemically skinned, and then exposed to a series of Ca^2+^-buffered solutions of increasing Ca^2+^ concentrations. Slow- and fast-twitch myofibers were classified by a strontium sensitivity test (see details in Materials and Methods).

**Figure 4:**
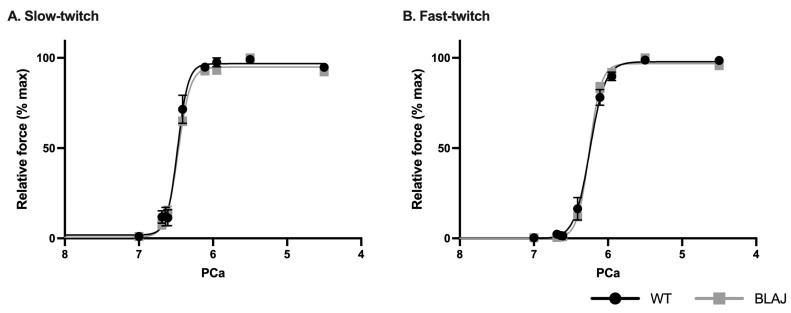
Force-power of Ca^2+^ (pCa) relationship of chemically skinned (**A**) slow- and (**B**) fast-twitch myofibers isolated from soleus and EDL muscles from normal WT and dysferlin-deficient BLAJ mice aged 3 months. There were no strain-specific differences for slow- or fast-twitch myofibers (classified by strontium sensitivity test) (*p* > 0.05). Data are expressed as percentage of maximum force, presented as mean ± SEM (*n* = 3–7).

Dysferlin deficiency did not impact the Ca^2+^-activation properties of these myofibers, with no differences between WT and BLAJ for maximum Ca^2+^-activated specific force (Table 2), nor Ca^2+^ sensitivity, including force-power of Ca^2+^ (pCa) curves (Figure 4) and measures of Hillslope coefficient, pCa_10_, pCa_50_, or pCa_90_ (*p* > 0.05; Table 2).

Note that similar isolated myofiber analyses were attempted with 10-month-old mice, however, the older BLAJ slow-twitch myofibers readily broke during the extraction procedure. Therefore, due to this fragility, this approach was abandoned for the older mice.

## 3. Discussion

This study investigated the differential impact of dysferlin deficiency on parameters related to the contractile function of intact, predominantly slow- and fast-twitch (soleus and EDL) skeletal muscles, by assessing the abundance of selected proteins related to Ca^2+^ regulation and glucose/glycogen metabolism, the glycogen content of muscles, and also the Ca^2+^-activation properties of contractile apparatus (i.e., myofilaments) using isolated chemically skinned slow- and fast-twitch myofibers.

### 3.1. Dysferlin Deficiency Differentially Alters the Levels of Ca^2+^ Handling and Glucose/Glycogen Metabolism Proteins in BLAJ Soleus and EDL Muscles at 10 Months of Age

**Figure 5:**
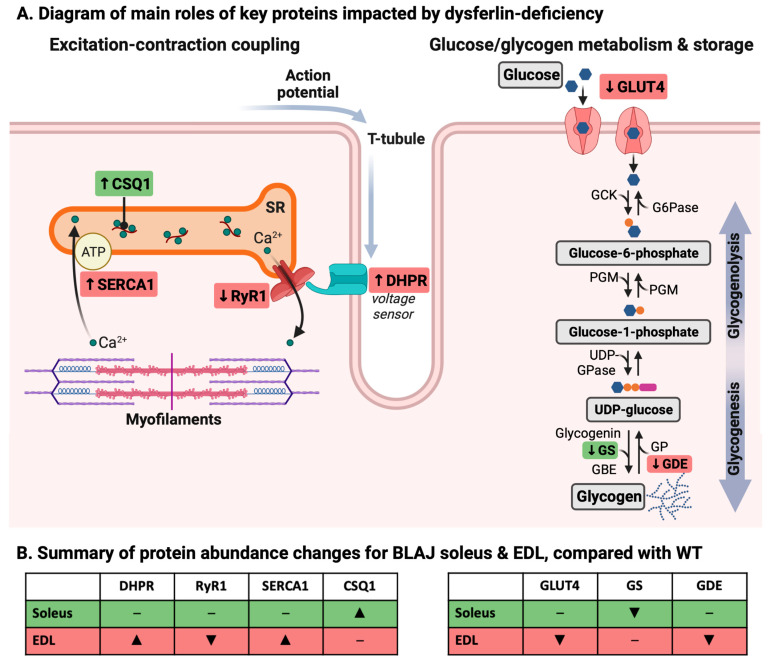
Summary diagram of myofiber-type specific differences in protein levels related to Ca^2+^ handling (i.e., excitation-contraction coupling) and glucose/glycogen metabolism for dysferlin-deficient BLAJ muscles. (**A**) Simplified diagram indicating location and direction of change (arrows) of affected proteins, for male BLAJ slow soleus (shown in green) and fast EDL (shown in red) muscles, relative to WT mice, aged 10 months (*n* = 1–6). (**B**) Summary emphasizing relative changes in these protein abundances (▲, ▼, **–** indicate an increase, decrease, or no change, respectively). Abbreviations: ATP: Adenosine triphosphate; Ca^2+^: Calcium; CSQ1: Calsequestrin 1; DHPR: Dihydropyridine receptor; G6Pase: Glucose-6-phosphatase; GBE: Glycogen branching enzyme; GCK: Glucokinase; GDE: Glycogen debranching enzyme; GLUT4: Glucose transporter 4; GP: Glycogen phosphorylase; GS: Glycogen synthase; PGM: Phosphoglucomutase; RyR1: Ryanodine receptor 1; SERCA1: Sarco/endoplasmic reticulum Ca^2+^-ATPase 1; SR: Sarcoplasmic reticulum; T-tubule: Transverse tubule; UDP-GPase: Uridylyltransferase-glucose pyrophosphorylase. Created with BioRender.com.

Striking differences were observed in the abundance of some Ca^2+^ handling and glucose/glycogen metabolism-associated proteins in muscles from 10-month-old BLAJ mice, compared with WT, that varied between the predominantly slow soleus and fast EDL muscles. The main roles of these impacted proteins are summarized diagrammatically in Figure 5. These protein level changes likely relate to published observations of dysf^−/*−*^ myofiber-type specific differences reported for function and metabolism, as discussed below.

#### 3.1.1. Ca^2+^-Handling Proteins

An increase in CSQ1 protein in BLAJ soleus muscle, compared with WT, supports our observation of faster contraction and relaxation for BLAJ soleus muscle at 10 months [26]. CSQ1 is a high-affinity Ca^2+^-buffering protein, with higher affinity for Ca^2+^ than the CSQ2 isoform (which is typically present in slow-twitch myofibers), hence increased abundance of CSQ1 will enhance the Ca^2+^-buffering capacity of the sarcoplasmic reticulum and may increase the rate of Ca^2+^ release and reuptake [28] and subsequent contraction and relaxation times.

In contrast, the BLAJ EDL had approximately five-fold more DHPR protein than WT, which may compensate for the extremely low RyR1 protein levels (undetectable in five of the six samples), or vice versa, in order to ‘maintain’ muscle function. Typically, DHPR proteins are associated with a single RyR tetramer. However, many RyR tetramers are not coupled to a DHPR. The increased DHPR protein content suggests an increase in the overall DHPR:RyR1 and hence enhanced EC coupling-induced Ca^2+^ release and submaximal force production [29,30]. Indeed, we reported increased force production (relative to maximal force) in BLAJ EDL muscles at some submaximal stimulus frequencies [26]. We now report that in those same muscles that showed an increase in force, there was an increased abundance of SERCA1 protein, suggesting that the EDL would have an enhanced capacity for fast relaxation as cytosolic [Ca^2+^] could be cleared faster. Contrary to this, however, we reported slowed relaxation in the BLAJ EDL [26]. Whilst not examined in these same samples due to difficulty with analyses, sarcolipin and phospholamban, which are small proteins that regulate SERCA activity warrant investigation [31] and may explain why there were no functional consequences of the increased SERCA1 protein content in the BLAJ EDL. Thus, a complexity of compensatory protein changes may help to maintain the function of these dysf^−/−^ BLAJ muscles.

Further, we cannot conclude that these altered levels of Ca^2+^-handling proteins are primarily responsible for the subtle contractile function differences and disrupted Ca^2+^ handling reported for dysf^−/−^ muscles since other factors have also been implicated. These factors include altered T-tubule structure [10,21] and dysferlin’s interaction with, and possible altered function of Ca^2+^-handling proteins including DHPR and RyR [19,22,23,32,33]. Therefore, further investigation of Ca^2+^ handling in dysf^−/−^ muscle is warranted to gain greater understanding of the loss of muscle function in dysferlinopathy and the potential broader impacts of Ca^2+^ dysregulation including muscle damage and inflammation [32,34,35].

#### 3.1.2. Glucose/Glycogen Metabolism Proteins

Our results indicating altered glycogen metabolism, as measured by the glycogen-associated proteins, fit with previous observations, albeit only seen in abstract form, of disturbed glucose metabolism in dysferlinopathy [24]. Lower glycogen synthase in the BLAJ compared with WT soleus suggests a decreased capacity to synthesize glycogen. However, as no strain differences for other glycogen-related proteins or for GLUT4 were evident, there may be little overall effect on glycogen metabolism in the dysf^−/−^ soleus muscle. This was supported by the similar glycogen content in soleus muscles from BLAJ and WT mice, when assessed by two-way ANOVA with EDL muscle. The regulation of glycogen synthase activity is complex [36] and the protein measurement is insufficient to determine activity. It is evident that the lower glycogen synthase did not translate to decreased glycogen content, and so this does not seem to provide a link to the increased reliance on lipids reported for BLAJ soleus muscles [12] or contribute to the delayed fatigue recovery reported for 10-month-old BLAJ soleus [26].

In the BLAJ EDL, the reduced GLUT4 abundance indicates that glucose uptake may be reduced. However, determining the functional relevance of this requires a full investigation of GLUT4 dynamics at the sarcolemma, which is technically difficult to achieve with quantitative outcomes. In addition, glycogen debranching enzyme protein was also reduced in BLAJ EDL compared with WT mice, which may limit glycogen utilization and would provide a negative feedback signal that results in reduced glucose uptake. Whilst there was no difference in the glycogen content of EDL muscles between BLAJ and WT, it was lower in EDL compared with soleus muscle of BLAJ mice. Since many proteins not included in our analyses can interact with, and influence glycogen utilization and synthesis, these preliminary data suggest that a deeper analysis of glycogen metabolism, in both slow and fast-twitch dysf^−/−^ muscles, is warranted.

We also measured the abundance of the enzyme cytochrome c oxidase subunit IV (COXIV), considered a marker of mitochondrial content [37], and protein levels were not altered in soleus and EDL muscles of these male BLAJ mice aged 10 months. Lipids are an alternative major fuel for muscle contraction, and there is a striking lipid-associated pathology in dysferlinopathy. While extensive analyses of lipid metabolism, lipidomics and associated gene expression have been described for young [12] and very old [38] BLAJ compared with WT mice, also of potential interest is quantification of levels of proteins regulating lipid metabolism in various BLAJ skeletal muscles.

### 3.2. Dysferlin Deficiency Does Not Disrupt Contractile Apparatus Ca^2+^ Activation in Mice at Three Months

To examine whether dysferlin deficiency also impacted the intrinsic Ca^2+^-activation properties of slow- and fast-twitch contractile apparatus, we used chemically skinned isolated myofibers from soleus and EDL muscles of WT and BLAJ mice aged three months. No strain-related differences were observed in the maximum Ca^2+^-activated force nor Ca^2+^ sensitivity of the contractile apparatus. We attempted similar isolated myofiber analyses with 10-month-old mice, however, the older BLAJ slow-twitch myofibers were fragile and difficult to excise. Therefore, this approach was abandoned for the older mice. Interestingly, when these myofibers broke, there were observable droplets released into the paraffin oil, which is not surprising due to the reported lipid droplet deposition in dysf^−/−^ slow oxidative (type 1) myofibers [9,10]. Therefore, these isolated myofiber results can only inform on the intrinsic impact of dysferlin deficiency on myofilament Ca^2+^ activation at three months of age, not potential age-related changes associated with disease progression. Nonetheless, these results rule out any direct effects of dysferlin deficiency on myofilament force production and indicate that altered contractile function of BLAJ muscles is not due to intrinsic differences in myofilament responses to Ca^2+^, and rather support the likely involvement of altered levels of key proteins associated with EC coupling and Ca^2+^ handling in dysferlinopathy-related contractile dysfunction.

## 4. Materials and Methods

### 4.1. Animals and Muscle Dissection

This study examined whole soleus and EDL muscles, as well as isolated slow and fast myofibers of male dysf^−/−^ BLAJ mice, compared with WT normal C57BL/6J. The intact whole soleus and EDL muscles for immunoblotting analyses came from mice aged 10 months (*n* = 6) and were contralateral muscles from the same mice used to investigate functional differences between these muscles [26]. Isolated myofibers for Ca^2+^-activation analyses were obtained from both soleus and EDL (pooled left and right) muscles of mice aged three months (*n* = 2). The mice were maintained at the Preclinical Animal Facility at the University of Western Australia, housed individually in cages with food and water, and maintained in a 12-h light/dark regime at 20–22 °C. All animal procedures were approved by the Animal Ethics and Experimentation Committee of UWA (RA/3/100/1436), in accordance with the guidelines of the National Health and Medical Research Council of Australia.

Mice were anesthetized using sodium pentobarbitone (40 mg/kg of body mass; intraperitoneal (IP)) and placed on a heated plate at 37 °C to maintain core body temperature. For protein quantification by immunoblotting, muscles were excised and snap-frozen, and for isolated myofiber experiments, the soleus and EDL muscles from both hind limbs were excised, blotted dry, and kept in paraffin oil at 5 °C until use. Mice were then euthanized by an overdose of pentobarbitone (IP). In both cases, additional tissues were snap-frozen for later analyses.

### 4.2. Western Immunoblotting to Quantify Various Proteins in Intact Soleus and EDL Muscles

The protein levels were quantified using cryosectioned slices of frozen soleus and EDL muscles using Western blotting procedures, as described in detail previously [39,40,41]. Proteins assessed included CSQ1, SERCA1, GLUT4, GS, glycogen branching enzyme (GBE), glycogen phosphorylase (GP), GDE, and COXIV as described by [40], with DHPR and RyR1 as described by [41]. In brief, proteins, including 4–5 amounts of a mixed muscle homogenate used as a calibration curve, were separated on 4–15% Criterion Stain Free gels (Bio-Rad, Hercules, CA). After transferring to nitrocellulose and a series of washes, including antibody probes, protein bands were visualized using West Femto chemiluminescent substrate (Thermo Scientific, Carlsbad, CA, USA), images collected, and densitometry performed using Chemidoc MP system and Image Lab version 5.2 (Bio-Rad, Hercules, CA, USA). Total protein on the gels was imaged prior to transfer (Stain Free imager, Bio-Rad) and Western blot signals of given proteins normalized to total protein with both total protein and protein of interest expressed relative to their respective calibration curves [39].

### 4.3. Glycogen Assay

Soleus and EDL muscles from BLAJ and WT animals (*n* = 6 each) were homogenized in physiological buffer (1:50 mass:volume) and then 0.14 µg/µL amyloglucosidase (A1602, Sigma-Aldrich) was added. All sample tubes were heated at 70℃ for 30 min and 60 µL glacial acetic acid added, followed by two units of peroxidase-glucose oxidase (PGO) enzyme (P7119, Sigma-Aldrich, Saint Louis, MO, USA) which had been prepared by dissolving one capsule in 1 mL o-Dianisidine/dihydrochloride (D3252, Sigma-Aldrich) and made to 50 mL volume with ddH_2_O (then kept in dark according to manufacturer’s instructions). Extracts were heated overnight at 55 ℃, following which 200 µL 4 M KOH was added and then 240 µL of PGO solution. A series of glucose standards were prepared using serial dilutions of a 1 mg/mL stock solution over the range 15–500 µg/mL glucose with a reagent blank of ddH_2_O and treated as per the samples from the PGO step onwards. After 5–10 min and within 30 min, the absorbance of samples and standards were read on a UV-VIS spectrophotometer at 450 nm and the glucose content of samples determined relative to the glucose calibration curve.

### 4.4. Measurement of Contractile Apparatus Ca^2+^-Activation Properties in Isolated Myofibers

**Figure 6:**
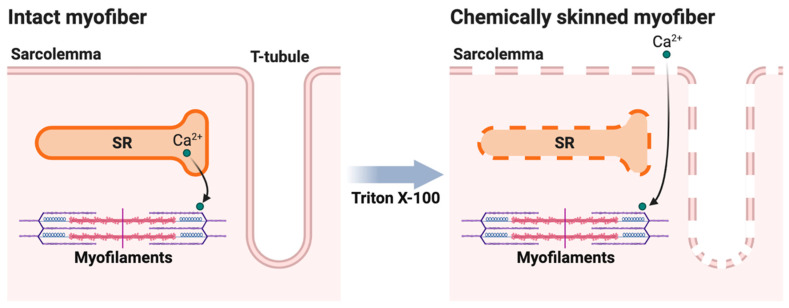
A schematic depiction of chemically skinning a myofiber. Chemical skinning by exposure to Triton X-100 disrupts all membrane structures of the myofiber: sarcolemma, transverse (T)-tubules, and sarcoplasmic reticulum (SR), and leaves only the contractile apparatus (myofilaments) intact. This bypasses the excitation-contraction coupling pathway and calcium (Ca^2+^) release from the SR, to allow Ca^2+^-activation properties of the myofilaments to be examined directly. Created with BioRender.com.

The isolation of single myofibers (from three-month-old mice) was performed with the muscle pinned out under paraffin oil, on a bed of Sylgard. Myofibers were isolated from the medial region of the respective soleus and EDL muscles. Following dissection, myofibers were chemically skinned through exposure to Triton X-100 for ~12 min, resulting in the disruption of all cellular membranous compartments, which destroys the endogenous Ca^2+^-handling system and leaves only the contractile apparatus functionally intact (see Figure 6 for a schematic diagram). This preparation primarily allows the Ca^2+^-activation properties of the contractile apparatus (i.e., myofilaments) in the isolated myofiber to be examined directly.

Each individual myofiber was mounted between a rigid pin, attached to the arm of a force transducer (model BAM4C, Scientific Instruments, Heidelberg, Germany), and a pair of fine forceps using braided silk thread. Force responses were recorded and analyzed using PowerLab data acquisition software (ADInstruments, Oxford, UK).

The Ca^2+^-activation properties of individual myofibers were determined by exposing chemically skinned myofibers to a series of Ca^2+^-buffered solutions of increasing Ca^2+^ concentrations from pCa (−log10[Ca^2+^]) > 8 to 4.5, prepared by mixing varying ratios of ‘relaxing solution’ and ‘maximal Ca^2+^-activating solution’. The relaxing solution was composed (in mM) of 126 K^+^, 36 Na^+^, 10.3 Mg^2+^, 50 EGTA, 90 HEPES, 8 ATP, 10 creatine phosphate, and 1 NaN_3_, with a pH of 7.10 ± 0.01. The maximal Ca^2+^-activating solution was comprised (in mM) similarly, but with 47.5 Ca^2+^ and 8.1 Mg^2+^. The solutions were prepared according to established methods [42]. Free Ca^2+^ concentrations were calculated using a previously determined apparent affinity constant for EGTA of 4.78 × 10^6^ [43].

Following exposure to the Ca^2+^ solutions, to identify each myofiber as either slow- (type 1) or fast-twitch (type 2), myofibers were exposed to two strontium (Sr^2+^)-EGTA-buffered solutions of known Sr^2+^ concentration, prepared by mixing different proportions of maximal Sr^2+^-activating solution with the relaxing solution A to achieve a power of Sr^2+^ of 5.5 (‘low’) and 4.5 (‘high’). Maximal Sr^2+^-activating solution was composed similarly to maximal Ca^2+^-activating solution but with 40 mM Sr^2+^ instead of Ca^2+^. Myofiber-type classification was determined by the ratio of the force responses to ‘low’ and ‘high’ pSr solutions, where slow-twitch myofibers (which are more sensitive to Sr^2+^ than fast-twitch myofibers) had a much higher ratio (~75–85%) compared with fast (~0–5%) [43].

Specific force (mN/mm^2^) was calculated as maximum Ca^2+^-activated force (mN) normalized to myofiber CSA (mm^2^) estimated from myofiber diameter measurements taken from a minimum of three sites along the myofiber. Myofiber CSA was assumed to be ellipsoidal and was approximated by the formula: CSA = (π × *a* × *b*)/4, where *a* is the major axis of the ellipse (taken as the largest diameter measurement), and *b* is the minor axis of the ellipse (smallest diameter measurement).

Ca^2+^-activated force responses were expressed as a percentage of maximal force and plotted as a function of pCa. Data were fitted with sigmoidal curves using GraphPad Prism (Version 9, GraphPad Software, San Diego, CA, USA), and the Ca^2+^-sensitivity properties: Hillslope coefficient (the maximum slope of the force-pCa curve, and an estimate of cooperative interactions between myofibrillar elements), and pCa_X_ (the amount of Ca^2+^ needed to produce ‘X’ amount of force) were derived.

### 4.5. Statistical Analyses

All data are presented as mean ± standard error of mean (SEM). Data were examined using separate one-way ANOVAs, two-way ANOVAs, Student’s *t*-tests, or non-parametric Mann-Whitney U tests where appropriate. Post hoc comparisons with Holm-Bonferroni corrections were conducted for each statistical test where appropriate, with statistical significance taken as *p* < 0.05. Statistical analyses were performed using Jamovi (Version 1.6).

## 5. Conclusions

This study, together with the results of Lloyd et al. [26] showing subtle myofiber-type specific differences in muscle function for the 10-month-old BLAJ soleus and EDL muscles (that were not fully accounted for by altered myofiber-type composition), implicates dysferlin deficiency in the disruption of EC coupling, potentially contributing to impaired muscle function in dysferlinopathy. These data indicate that further investigation into the impact of dysferlin deficiency on proteins related to Ca^2+^ handling and metabolism in the context of muscle contraction is of interest. Moreover, our findings related to altered EC coupling and glucose metabolism (as a fuel to provide energy for contraction), strongly indicate distinct intrinsic myofiber-type specific effects of dysferlin deficiency with important implications for a more precise understanding of the molecular basis of the disease. The marked myofiber-type differences between BLAJ slow soleus and fast EDL muscles, further emphasize the potential importance of myofiber-type composition in different muscles for the manifestation of dysferlinopathy.

## Figures and Tables

**Table 1:** Body mass and relevant tissue characteristics for normal wild-type (WT) and dysferlin-deficient BLAJ mice aged 3 and 10 months. Data analyzed by Student’s *t*-test: *** BLAJ vs WT (*p* < 0.001), except for 3-month body mass data that were analyzed by Mann-Whitney U test. Data are presented as mean ± standard error of mean (SEM). For isolated myofibers from 3-month-old mice, values in brackets indicate the number of myofibers analyzed. Data for 10-month mice were previously reported by Lloyd et al. [26] (CC BY 4.0). Abbreviations: BM: body mass; CSA: cross-sectional area; EDL: extensor digitorum longus.

	3 Months		10 Months	
	WT (*n* = 2)	BLAJ (*n* = 2)	WT (*n* = 8)	BLAJ (*n* = 9)
**Body mass (g)**	25.7 ± 0.4	24.1 ± 0.9	33.3 ± 0.7	34.4 ± 0.6
**Slow-twitch myofiber CSA (μm^2^)**	927 ± 46 (3)	1047 ± 58 (6)		
**Fast-twitch myofiber CSA (μm^2^)**	1205 ± 207 (6)	922 ± 121 (7)		
**Soleus mass (mg/gBM)**			0.33 ± 0.01	0.41 ± 0.01 ***
**EDL mass (mg/gBM)**			0.40 ± 0.01	0.40 ± 0.01

**Table 2:** Ca^2+^-activation properties of chemically skinned slow- and fast-twitch myofibers isolated from soleus and EDL muscles from normal WT and dysferlin-deficient BLAJ mice aged 3 months (*n* = 2). There were no strain-specific differences for slow- or fast-twitch myofibers (classified by strontium sensitivity test) (*p* > 0.05). Data are presented as mean ± SEM and analyzed by two-way ANOVA. Number of isolated myofibers analyzed indicated by *n* values.

	Slow Myofibers		Fast Myofibers	
	WT (*n* = 3)	BLAJ (*n* = 6)	WT (*n* = 6)	BLAJ (*n* = 7)
**Maximum specific force (mN/mm^2^)**	371 ± 6	318 ± 19	218 ± 24	312 ± 65
**Hillslope**	5.71 ± 0.99	5.05 ± 0.47	4.62 ± 0.37	5.16 ± 0.30
**pCa_10_**	6.65 ± 0.03	6.65 ± 0.03	6.45 ± 0.03	6.43 ± 0.01
**pCa_50_**	6.48 ± 0.03	6.47 ± 0.01	6.24 ± 0.03	6.25 ± 0.01
**pCa_90_**	6.30 ± 0.04	6.24 ± 0.01	6.02 ± 0.05	6.05 ± 0.02

## Data Availability

Data available on request.
